# Widespread detection of highly pathogenic H5 influenza viruses in wild birds from the Pacific Flyway of the United States

**DOI:** 10.1038/srep28980

**Published:** 2016-07-06

**Authors:** S. N. Bevins, R. J. Dusek, C. L. White, T. Gidlewski, B. Bodenstein, K. G. Mansfield, P. DeBruyn, D. Kraege, E. Rowan, C. Gillin, B. Thomas, S. Chandler, J. Baroch, B. Schmit, M. J. Grady, R. S. Miller, M. L. Drew, S. Stopak, B. Zscheile, J. Bennett, J. Sengl, Caroline Brady, H. S. Ip, E. Spackman, M. L. Killian, M. K. Torchetti, J. M. Sleeman, T. J. Deliberto

**Affiliations:** 1 US Department of Agriculture–National Wildlife Research Center, Fort Collins, Colorado, USA; 2 US Geological Survey–National Wildlife Health Center, Madison, Wisconsin, USA; 3 Washington Department of Fish and Wildlife, Olympia, Washington, USA; 4 Oregon Department of Fish and Wildlife, Corvallis, Oregon, USA; 5 USDA Wildlife Services, Salem, Oregon, USA; 6 USDA Wildlife Services, Sacramento, California, USA; 7 USDA Veterinary Services, Fort Collins, Colorado, USA; 8 Idaho Department of Fish and Game, Caldwell, Idaho, USA; 9 USDA Wildlife Services, Boise, Idaho, USA; 10 USDA Wildlife Services, Salt Lake City, Utah, USA; 11 USDA Wildlife Services, Reno, Nevada, USA; 12 California Waterfowl Association, Roseville, California, USA; 13 USDA ARS Southeast Poultry Research Laboratory, Athens, Georgia, USA; 14 US Department of Agriculture, Ames, Iowa, USA.

## Abstract

A novel highly pathogenic avian influenza virus belonging to the H5 clade 2.3.4.4 variant viruses was detected in North America in late 2014. Motivated by the identification of these viruses in domestic poultry in Canada, an intensive study was initiated to conduct highly pathogenic avian influenza surveillance in wild birds in the Pacific Flyway of the United States. A total of 4,729 hunter-harvested wild birds were sampled and highly pathogenic avian influenza virus was detected in 1.3% (n = 63). Three H5 clade 2.3.4.4 subtypes were isolated from wild birds, H5N2, H5N8, and H5N1, representing the wholly Eurasian lineage H5N8 and two novel reassortant viruses. Testing of 150 additional wild birds during avian morbidity and mortality investigations in Washington yielded 10 (6.7%) additional highly pathogenic avian influenza isolates (H5N8 = 3 and H5N2 = 7). The geographically widespread detection of these viruses in apparently healthy wild waterfowl suggest that the H5 clade 2.3.4.4 variant viruses may behave similarly in this taxonomic group whereby many waterfowl species are susceptible to infection but do not demonstrate obvious clinical disease. Despite these findings in wild waterfowl, mortality has been documented for some wild bird species and losses in US domestic poultry during the first half of 2015 were unprecedented.

Avian influenza viruses (AIV) have been identified in more than 100 wild bird species^1^ and *Anseriformes* (ducks, geese, and swans) and *Charadriiformes* (gulls, terns, and waders) are considered to be the primary wild reservoirs for these viruses. Wild birds infected with AIVs often display no signs of clinical disease, although this can vary widely based on the bird species and virus subtype involved. The classification of AIVs as high pathogenic or low pathogenic is based on the lethality of the virus in chickens^2^. Highly pathogenic avian influenza viruses (HPAIV) often cause substantial mortality in chickens, other domestic birds, and in some cases, wild avian species, although HPAIVs are not thought to persist or circulate widely in wild birds^3^. Low pathogenic H5 and H7 influenza subtypes have the ability to evolve into HPAIVs that are lethal in domestic poultry^1^,^4^,^5^,^6^,^7^ and so these subtypes are of particular concern because of their potential to cause large scale avian mortality and economic losses.

In early 2014, the Republic of Korea reported the occurrence of an H5 clade 2.3.4.4 HPAIV in domestic poultry and wild waterfowl^5^. Although the H5N8 virus was novel, the H5 gene of this virus was determined to be a descendent of the highly pathogenic H5N1 virus first detected in China in 1996 (Goose/Guangdong/96 lineage)^8^. Between January and July of 2014 the H5N8 HPAIV infected domestic poultry at 212 farms in the Republic of Korea^4^,^8^,^9^ and was detected in numerous local wild birds (including both live and dead waterfowl)^10^. Detections in domestic poultry in China, Japan, and Taiwan soon followed^11^.

In autumn of 2014, H5N8 HPAIV was detected in a hunter-harvested Eurasian wigeon (*Anas penelope*) in northern Russia, more than 3000 km from the Republic of Korea but within the known breeding range of this species^12^. Soon after this detection, HPAIV H5N8 was reported in either commercial poultry or wild bird species in Germany, the Netherlands, and the United Kingdom^13^,^14^,^15^. Genetic differences observed in viruses obtained from the European outbreaks, along with a lack of connection between the affected premises, suggest that, in most cases, there were multiple introductions of the virus rather than a single introduction followed by transmission between farms^16^.

In late November 2014, a reassortant HPAIV H5N2 subtype–with 5 gene segments similar to the Republic of Korea H5N8 and 3 gene segments from North American wild bird AIV lineages–was found in a British Columbia, Canada poultry operation. Between November and December the virus spread to 11 poultry farms in British Columbia and in December was found in a wild northern pintail (*Anas acuta*) in the United States. At the same time, and in close proximity to the positive pintail, a H5N8 that was >99% similar to the H5N8 HPAIVs detected in Asia and Europe was found in a captive gyrfalcon (*Falco rusticolos*) that had fed on wild duck meat^17^. This group of H5 clade 2.3.4.4 viruses—including European, Russian, Korean, Japanese, Chinese, and North American viruses—has been characterized as intercontinental group A (icA)^9^.

The detection of these viruses on a global scale, combined with multiple detections in both live and dead wild birds, led to increased speculation that wild birds were involved in the movement of these icA H5 viruses between continents^9^,^10^,^15^,^18^,^19^. Following the detection of HPAIV in Canada and Washington, a multi-agency study was initiated to examine wild birds throughout the Pacific Flyway. This monitoring effort focused on sample collection from hunter-harvested wild birds in the Pacific Flyway of the United States combined with enhanced wild bird morbidity and mortality surveillance. The goals of this effort were (1) to identify the distribution of the Eurasian H5 clade 2.3.4.4 viruses in wild birds throughout the Pacific Flyway of the United States, which could aid in determining the risk these viruses posed to US poultry producers and wild bird populations, and (2) to better understand what subtypes and what reassortant viruses were present in the US.

## Results

### Wild Bird Sampling

**Figure 1:**
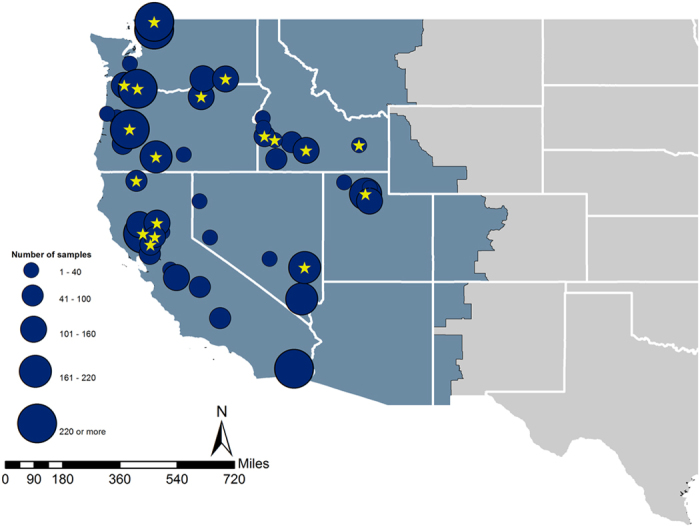
Circle diameter represents number of wild bird samples collected in that county; star denotes that at least one icA H5 clade 2.3.4.4 virus was detected from that set of samples. Background color represents the administrative boundary of the Pacific Flyway. Maps were produced using ArcGIS software by Esri, version 10.3 (http://desktop.arcgis.com).

From December 20, 2014 through February 1, 2015 a total of 4,729 oral and cloacal swab were opportunistically collected from hunter-harvested birds. These samples were obtained from 6 states in the Pacific Flyway (Fig. 1, Table 1), with 33% of samples coming from California, 9% from Idaho, 9% from Nevada, 19% from Oregon, 7% from Utah, and 23% from Washington. Sample locations were spread across 45 counties and nearly 100 sample locations. A total of 33 species were sampled, the majority of which were dabbling ducks, known reservoirs for AIVs^6^,^20^.

Of the samples tested, 469 (mean prevalence = 9.9%, 95% CL = 9.1–10.8) were influenza A positive (Table 2) as detected by real-time reverse transcriptase polymerase chain reaction (rRT-PCR). These 469 influenza A positive samples came from 15 different species (Table 2). Of the influenza A positive samples, 113 (mean prevalence = 2.4%, 95% CL = 2.0–2.9) tested positive for H5 by rRT-PCR; 55 tested positive for H7 (mean prevalence = 1.2%, 95% CL = 0.87–1.5). All H7 positive samples tested for pathogenicity (n = 35), were determined to be low pathogenicity viruses. Of the H5 positive samples, 63 were positive by molecular assays, including a highly specific Eurasian H5 clade 2.3.4.4 assay (D. Suarez, unpublished), for H5 icA viruses (mean prevalence out of 4,729 samples = 1.3%, 95% CL = 0.98–1.7). No virus was isolated from 22 of the 63 positive samples and so the neuraminidase (NA) subtype was not determined. Isolates (n = 41) from the remaining positives were identified as H5N2 (n = 19), H5N8 (n = 19), H5N1 (n = 3). There were also two possible mixed infections with an H5 clade 2.3.4.4 positive and a LPAIV; however, the subtype was not definitively determined prior to publication. Species that were HPAIV positive were American green-winged teal (*Anas crecca*, n = 4), American wigeon (*Anas americana*, n = 31), Canada goose (*Branta canadensis*, n = 1), gadwall (*Anas strepera*, n = 1), mallard (*Anas platyrhynchos,* n = 15), northern pintail (n = 5), northern shoveler (*Anas clypeata*, n = 3), and wood duck (*Aix sponsa*, n = 3)^21^.

There were no HPAIVs detected in the US during a previous wild bird surveillance effort that occurred from 2006–2011^22^,^23^ so a direct comparison between wild bird HPAIV prevalence before and after the arrival of the Eurasian H5 clade 2.3.4.4 cannot be made. We can, however, compare the H5 virus prevalence between the two time periods. The H5 prevalence in mallards and wigeons (two of the most commonly sampled dabbling ducks in both surveillance efforts) sampled between December 2014 and February 2015 in the Pacific Flyway reveal that prevalence of H5 viruses was significantly higher (F = 44.30, p < 0.0001) than in 2006–2011. The odds of these waterfowl being infected with an H5 are 11.9 times higher (95% CL 5.7–24.7) than they were from 2006–2011, with 3.5% (78/2186, 95% CL = 2.8–4.4) of mallards and wigeons currently testing positive for H5, versus 0.3% (8/2586, 95% CL = 0.1–0.6) during the previous surveillance effort. In contrast, prevalence of H7 viruses did not significantly differ (F = 3.16, p = 0.08) between mallards and wigeons sampled and tested for H7 from 2006–2011 and mallard and wigeons sampled in this current dataset (0.17%, 5/2890, 95% CL = 0.02–0.3 versus 0.46%, 10/2186, 95% CL = 0.1–0.7).

### Morbidity/Mortality Sampling

From December 1, 2014 to February 28, 2015 a total of 150 (December = 83, January = 50, February = 17) sick or dead birds from the state of Washington were tested for HPAIV at the U.S. Geological Survey’s National Wildlife Health Center. Four morbidity and mortality events involved multiple birds and accounted for 61 sick or dead birds, including 41 carcasses obtained from the index location (Wiser Lake, Whatcom County, Washington), and three other separate events with 5, 7, and 8 sick or dead birds examined. All remaining submissions were individual sick or dead bird investigations. Of the 150 sick or dead birds, 41 only had swab samples submitted. Full carcasses were received for the remaining 109 submissions (Table 3). Six out of 97 waterfowl samples (6.2%) were icA H5 positive (Canada goose, northern pintail, mallard, American wigeon) and 4 out of 35 raptor samples were icA positive (11.4%). Positive raptor species included peregrine falcon (*Falco peregrinus*), Cooper’s hawk (*Accipiter cooperii*), and red-tailed hawk (*Buteo jamaicensis*). No HPAIV positive samples were identified from other water birds and no HPAIV positive samples were identified when only swab samples were submitted. Five of the icA H5 detections were made in waterfowl found dead at the index location from the ongoing morbidity and mortality event^17^. In addition 4 individual raptors found dead in 4 different Washington counties (Whatcom, Skagit, Grays Harbor, and Benton), and a single Canada goose found sick in Jefferson County, WA were icA H5 positive. None of the individual raptors or the single Canada goose that tested icA H5 positive were associated with morbidity or mortality events. H5N2 was detected in 7 individual birds and H5N8 was detected in 3 birds (Table 4).

**Figure 2:**
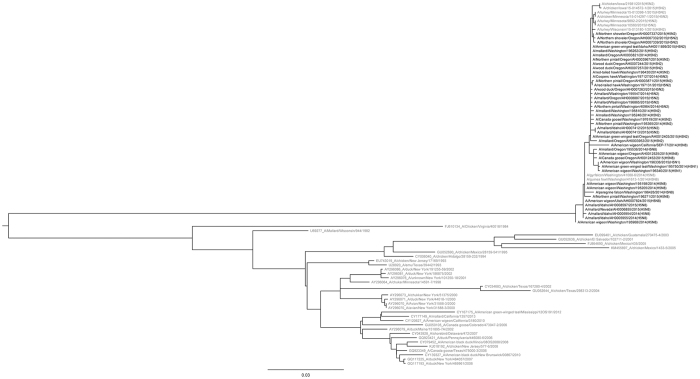
Phylogenetic comparison of hemagglutinin genes from highly pathogenic avian influenza A (H5N2, H5N8, and H5N1) detected in wild birds from the United States Pacific Flyway. Sequences were aligned using Clustal W. Evolutionary analyses were conducted in MEGA6 and the evolutionary history was inferred by using the Maximum Likelihood method based on the Hasegawa-Kishino-Yano (HKY) model^43^,^44^. The tree with the highest log likelihood is shown. Bolded samples were from wild birds sampled in the Pacific Flyway. The HA analysis involved 85 nucleotide sequences, 42 of which were from samples collected during this surveillance effort.

**Figure 3:**
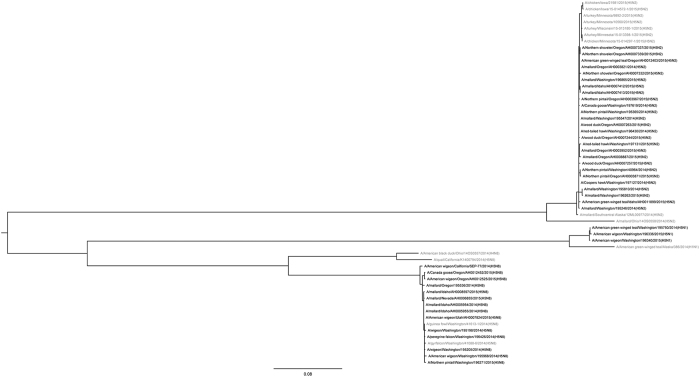
Phylogenetic comparison of neuraminidase genes from highly pathogenic avian influenza A (H5N2, H5N8, and H5N1) detected in wild birds from the United States Pacific Flyway. Sequences were aligned using Clustal W. Evolutionary analyses were conducted in MEGA6 and the evolutionary history was inferred by using the Maximum Likelihood method based on the Hasegawa-Kishino-Yano (HKY) model^43^,^44^. The tree with the highest log likelihood is shown. Bolded samples were from wild birds sampled in the Pacific Flyway. Analysis of the NA gene involved 57 nucleotide sequences, 43 of which were from samples collected during this surveillance effort.

Phylogenetic analyses of the H5 clade 2.3.4.4 viruses detected as part of both the hunter-harvested surveillance effort and the morbidity and mortality surveillance reveal a high degree of relatedness (Figs 2 and 3). The H5N2, H5N8, and H5N1 viruses were co-circulating in wild birds sampled in the Pacific Flyway during the surveillance effort.

## Discussion

The goals of this monitoring effort were to determine if Eurasian clade 2.3.4.4 viruses, newly introduced to North America, were present in US Pacific Flyway wild bird populations and if so, to better understand the extent to which they were circulating. The study was motivated by both the rapid spread of these H5 viruses in Asia and Europe and by the detection of these viruses in Canada^8^,^9^,^24^. Eurasian clade 2.3.4.4 viruses were detected in 1.3% (63 of 4729) of hunter-harvested waterfowl sampled in the Pacific Flyway. Three different neuraminidase subtypes were detected, all of which were part of the H5 clade 2.3.4.4 lineage, including the wholly Eurasian lineage H5N8, as well as Eurasian-North American reassortants H5N2 and H5N1; all of which are >99% similar^17^ across Eurasian gene segments to other icA H5 HPAIVs^17^,^25^. The reassortant H5N2 (19/41, 46.3%), and the H5N8 (19/41, 46.3%) were equally prevalent in hunter-harvested birds. Three hunter-harvested samples also tested positive for the reassortant H5N1 (3/41, 7.3%). H5N2 (n = 7) and H5N8 (n = 3) were detected in samples from sick or dead birds.

The isolation of multiple reassortant viruses in wild birds is not surprising given the well-known occurrence of genetic recombination in influenza viruses, which occurs when more than one virus infects the same host cell. The segmented genome of influenza viruses allows segments from different viruses to be traded and in this case, introduction of the Eurasian H5 viruses into the North American influenza gene pool appears to have generated multiple novel subtypes (Eurasian-American lineage H5N2 and H5N1)^17^,^25^. Taiwan also identified novel reassortants after H5 clade 2.3.4.4 viruses were initially detected^26^. Although it is not surprising to see reassortant viruses, their emergence in what is believed to be a relatively short time frame is intriguing. de Vries *et al*.^11^ noted that this emergence of new H5 combinations is unprecedented in H5N1 evolutionary history. In North America and in other places such as China and Taiwan^17^, rapid reassortment of clade 2.3.4.4 viruses may be facilitated through encounters with a large and diverse population of LPAIVs in wild birds, although this pattern was not necessarily seen in Europe where the association of this novel virus with wild bird populations does not appear to have spawned similar reassortant events in the same relatively short time frame.

Interestingly, more than half of the H5 detections (63/113 samples, 60%), and 13% (63 of 469) of all influenza A detections from hunter-harvest surveillance samples were Eurasian H5 HPAIVs, suggesting that the H5 clade 2.3.4.4 viruses were a substantial part of the influenza A gene pool in US wild bird populations during the winter of 2014–2015. Wild bird influenza surveillance efforts carried out from 2006–2011^22^,^23^ found a significantly lower prevalence of H5 viruses in winter samples in the two most commonly sampled species (mallards and wigeon) from the Pacific Flyway when compared to this current effort. Samples collected from American wigeons accounted for almost half of all icA H5 detections (31 of 63) in wild waterfowl. American wigeons (n = 777) were the species most frequently infected with icA H5 despite mallards being the most frequently sampled species (1,410 mallard samples collected, 15 HPAIV detections). The high infection prevalence of icA H5 viruses detected during this wild bird sampling effort likely at least partially reflects the introduction of a new virus into a naïve population of birds that had no previous exposure to clade 2.3.4.4 viruses; however, it is unknown if these viruses will continue to circulate at high levels over time. In general, it is thought that HPAIV viruses are not maintained in wild bird populations. This is primarily based on HPAIV having rarely been isolated from wild birds prior to this emergence, even though prior research has found H5N1 outbreaks in domestic poultry to closely associate with known wild bird migration routes^27^. Although this effort documented the widespread occurrence of HPAIV in wild bird populations, the possibility of long-term persistence is unknown. Our understanding of HPAIV dynamics in wild birds in still limited despite a substantial increase in research on influenza over the last decade and this highlights the need for continued, consistent monitoring of HPAIV dynamics in wild birds over time.

Detection of icA H5 HPAIV in apparently healthy wild birds^10^, combined with laboratory studies in avian species that demonstrate low morbidity and mortality, reinforces the likely role of wild waterfowl in the emergence and geographic spread of these viruses^4^,^5^,^19^. Wild waterfowl infected with icA H5 viruses are likely capable of moving these viruses short distances, and possibly even long distances, along migration routes; however, the length of time that an infected bird sheds an influenza virus, which is typically up to 10–14 days in experimental infection with other goose/Guangdong/96 lineage isolates in ducks^28^,^29^, also plays a role in the likelihood of virus introduction via long-distance migration.

Despite the widespread detection of Clade 2.3.4.4 H5 viruses in wild birds sampled during this effort, only two commercial poultry facilities were infected in the Pacific Flyway of the US during 2015, both with icA H5N8. Eight backyard poultry flocks were also infected in the Pacific Flyway (one with icA H5N8, the rest with icA H5N2). This is in contrast to the icA H5 outbreaks seen several months later in commercial poultry facilities in the Central and Mississippi Flyways. By June 2015, there were 213 icA H5N2 outbreaks in commercial and backyard poultry within the Central and Mississippi Flyways^30^,^31^. The mechanisms responsible for the different scale of commercial poultry outbreaks in the Pacific Flyway compared to the other flyways is largely unknown, although commercial outbreaks were shown to be a combination of both independent/point source introductions along with common source or lateral spread^30^. The latter would be independent of wild bird influenza dynamics. Furthermore, the wholly Eurasian H5N8 virus accounted for almost 40% of HPAIVs in wild birds, but only 4 of the 223 documented domestic poultry outbreaks in the US between December of 2014 and June of 2015 were caused by H5N8^30^. Laboratory studies suggest that these viruses were well-adapted to the waterfowl host, with experimentally infected mallards remaining asymptomatic while easily transmitting the virus to conspecifics. This is in contrast to the less efficient transmission to contacts seen in infected chickens^32^. These differences may play a role in the different patterns seen in wild birds versus domestic poultry. Although the wild bird data reported here were collected between December 2014 and February 2015 in the Pacific Flyway of the US, most of the domestic poultry outbreaks in the U.S. occurred February–May 2015 in the Midwestern US, representing differences in both time and space and making direct comparisons difficult. Between March and June, 2015, US poultry producers lost nearly 50 million birds to these outbreaks^31^.

This effort focused on waterfowl because they are considered a primary influenza A virus reservoir, and while these data suggest that some waterfowl species are not negatively impacted by infections with Eurasian H5 clade 2.3.4.4 viruses, it appears that other wild species may suffer severe morbidity and mortality. The first detection of icA reassortant H5N2 in wild birds in North America was obtained during a waterfowl mortality event at Wiser Lake, Whatcom County, Washington; although the cause of the mortality event was attributed to aspergillosis with the icA H5 infection reported as a secondary finding^17^. Concurrent to this waterfowl mortality event, a captive-reared gyrfalcon died from icA H5N8 after being fed wild wigeon from the same area^17^. From December through February, morbidity and mortality testing of wild birds in Washington increased; 150 additional waterfowl, other water birds, and raptors were tested as part of this effort. From these, icA H5 was detected in 6 waterfowl, (5 were ducks from Wiser Lake, the index location and included the northern pintail reported by Ip *et al*.^17^ and one from a Canada goose in a second county). Four raptors from 4 different counties were also positive and represented 11% of all raptors (n = 35) tested. Lesions in the lung and heart consistent with HPAI infection were observed in all four raptors. The cause of death in the five ducks found dead at the index location was determined to be aspergillosis with a HPAI non-lethal co-infection. The cause of death in the single Canada goose is suspected to be due to HPAI, but the role of HPAI infection was difficult to interpret due to freeze artifact and a co-bacterial infection in the lungs. Other sick and dead Canada geese and raptors testing positive for the icA H5 viruses have been reported in other states, suggesting they may be good targets for morbidity and mortality surveillance to identify presence of icA H5 viruses.

This surveillance effort that began immediately upon reports of HPAIV detection in British Columbia allowed early detection of clade 2.3.4.4 viruses in the US wild bird populations. This rapid response to collect and test wild bird samples offered an unprecedented opportunity to better understand the dynamics of a novel virus introduced into a naïve wild bird population with its own endemic and diverse influenza A gene pool. Passive surveillance of apparently healthy hunter-harvested wild birds was used in conjunction with morbidity and mortality surveillance and both methodologies provided valuable data. Continued surveillance and characterization of influenza A in wild birds is suggested in order to monitor virus evolution, to understand risk pathways for introduction, and to assess the emergence of mutations that may be relevant for veterinary and public health^15^.

## Methods

### Sample Areas

Wild bird sample collection focused on the Pacific Flyway of the US. Waterfowl and water bird migration in North America generally consists of north-south seasonal movements between breeding grounds and wintering areas and while the flyway boundaries are not biologically fixed or sharply defined, the flyways represent the prominent movement pathways of migratory waterfowl^22^. Band-recovery data on waterfowl species of interest show that migratory waterfowl move throughout this flyway. Ten priority sampling areas were chosen in the Pacific Flyway. These areas were chosen based on previous research that identified geographic clusters of low pathogenic AIV in wild birds^23^,^33^ and known high concentration wintering areas for waterfowl in the Pacific Flyway. Sample areas roughly corresponded to a broad watershed scale. Sample sizes were based on 95% confidence in detecting 1 positive bird out of a population of 10,000 or more birds^34^. Expected prevalence was 1% based on previously published values of LPAIV^23^, and sensitivity and specificity of the diagnostic assays was 86.3% and 100% respectively (Janice Pedersen, personal communication).

### Sample Collection

More than 99% of samples came from hunter-harvested waterfowl and <1% came from birds removed as part of permitted wildlife damage management activities. Sampling of hunter-harvested birds was voluntary on the part of waterfowl hunters and birds were either swabbed immediately in the field or shortly after if hunters called agency field personnel to arrange sampling. Samples collected at hunter check stations were collected in accordance with the guidelines and regulations set forth by the United States Fish and Wildlife Service (USFWS) and with the permission of participating hunters. For most birds, separate swabs were used to collect an oropharyngeal and cloacal sample. The two swabs were placed in a single uniquely labeled cryovial containing 4 ml of viral transport media (VTM) or 3 ml of brain-heart infusion media (BHI). In some cases, waterfowl carcasses were damaged and in those instances only one swab sample was collected. In 4 cases a single swab was used to first swab the oropharyngeal cavity and then the cloacal cavity before being placed in the VTM. Used sample vials were held at 4 °C for up to 48 hours or were placed in liquid nitrogen vapor and frozen before being shipped overnight to a reference laboratory.

#### Morbidity and Mortality Sampling

Between December 4, 2014 and Feb 28, 2015 we obtained swab samples or whole carcasses from waterfowl, other water birds, and raptors found sick and dead in Washington State. Collection protocols for wild bird morbidity and mortality events were approved under USFWS permit #MB084762-1. Birds that were swabbed only had a single combined oral and cloacal swab submitted to the U.S. Geological Survey’s National Wildlife Health Center for avian influenza testing as described previously (for the live bird surveillance). In cases where whole carcasses were submitted, separate tracheal and cloacal swabs samples were obtained at minimum. Additional tissues tested could include trachea, lung, combined trachea–lung, brain, heart, and others, as identified by pathologists conducting a cause of death necropsy on the submitted carcass and varied by individual case. Tissues were processed and tested as previously described for hunter-harvest surveillance swabs.

### Laboratory Analyses

Testing and analyses were conducted at laboratories in the National Animal Health Laboratory Network (NAHLN) and at the Southeast Poultry Research Laboratory USDA-ARS (SEPRL). Presumptive positive findings were confirmed at the National Veterinary Service Laboratories-USDA-APHIS in Ames, Iowa, which is the US reference laboratory for avian influenza. Samples were initially screened by rRT-PCR utilizing a test which targets the influenza matrix (M) gene using the USDA standard procedure (SOP-AV-0001)^35^,^36^. Further testing on M-gene presumptive samples was conducted using H5 and H7 subtype rRT-PCR assays as a general surveillance tool^36^. A highly specific H5 IcA rRT-PCR (D. Suarez, personal communication) was also run on M gene positive samples to detect the Eurasian H5 clade 2.3.4.4 gene to distinguish IcA positive samples from those with the North American H5 gene. Virus isolation in embryonated chicken eggs was attempted on M-gene positive samples by standard methods^37^,^38^. Subtypes were identified by either serological assays (hemagglutination inhibition assay, neuraminidase inhibition assay) on isolates, or by gene sequencing on swab material or isolates using standard methods^39^,^40^,^41^. The pathotype classification was inferred from the HA gene proteolytic cleavage site sequence as defined by the World Organization for Animal Health (OIE)^42^. Select viruses were processed for *in vivo* pathotyping in specific pathogen free chickens at the NVSL in accordance with OIE guidelines^42^.

### Analyses

Prevalence and 95% confidence limits were calculated using an exact binomial calculation. Comparisons between H5 prevalence during a prior wild bird surveillance effort and this current surveillance used a general linearized model with a binary distribution and a logit link function. Odds ratios were calculated using a Tukey-Kramer adjustment. All analyses were run in SAS (SAS v.9.2, Cary, North Carolina, USA).

Genetic sequences were assembled using Clustal W. Sequences were only available from a subset of samples. All positions containing gaps and missing data were eliminated. Evolutionary analyses were conducted in MEGA6^43^. The evolutionary history was inferred by using the Maximum Likelihood method based on the Hasegawa-Kishino-Yano (HKY) model^44^. The tree with the highest log likelihood is shown. The percentage of trees in which the associated taxa clustered together is shown next to the branches. Initial trees for the heuristic search were obtained by applying the Neighbor-Joining method to a matrix of pairwise distances estimated using the Maximum Composite Likelihood (MCL) approach. A discrete Gamma distribution was used to model evolutionary rate differences among sites. Analysis of the HA gene involved 85 nucleotide sequences; analysis of the NA gene involved 57 nucleotide sequences.

## Additional Information

**How to cite this article**: Bevins, S. N. *et al*. Widespread detection of highly pathogenic H5 influenza viruses in wild birds from the Pacific Flyway of the United States. *Sci. Rep.* **6**, 28980; doi: 10.1038/srep28980 (2016).

## Figures and Tables

**Table 1:** Date of first H5 clade 2.3.4.4 sample collection from hunter-harvested wild birds, by state, along with number of HPAIV positives collected between December 2014 and February 2015.

**State**	**Date of First HPAIV Collection**	**Number of Wild Birds Sampled**	**Total HPAIV Positives**
Oregon	December 20, 2014	888	17
Idaho	December 22, 2014	413	6
Washington	December 23, 2014	1101	8
California	December 28, 2014	1563	30
Utah	January 2, 2015	350	1
Nevada	January 23, 2015	414	1
Total		4729	63

**Table 2:** Hunter-harvested wild bird surveillance rRT-PCR and highly pathogenic avian influenza virus (HPAIV) results for avian influenza matrix gene and hemagglutinin subtypes H5 and H7, Pacific Flyway, December 2014 through February 1st, 2015.

**Species**	**n**	**rRT-PCR result**			**HPAIV icA Positive**
		**Matrix Pos**	**H5 Pos**	**H7 Pos**	
Mallard, *Anas platyrhynchos*	1410	163 (11.5%)	37 (2.6%)	9 (0.6%)	15
Northern shoveler, *Anas clypeata*	555	82 (14.7%)	11 (1.9%)	18 (3.2%)	3
Green-winged teal, *Anas crecca*	724	75 (10.3%)	6 (0.8%)	22 (3.0%)	4
American wigeon, *Anas americana*	777	66 (8.4%)	39 (5.0%)	1 (0.1%)	31
Northern pintail, *Anas acuta*	460	43 (9.3%)	7 (1.5%)	2 (0.4%)	5
Cinnamon teal, *Anas cyanoptera*	67	12 (17.9%)	0	1 (1.4%)	0
Wood duck, *Aix sponsa*	27	8 (29.6%)	6 (22.2%)	0	3
Gadwall, *Anas strepera*	185	5 (2.7%)	3 (1.6%)	1 (0.5%)	1
Canvasback, *Aythya valisineria*	68	4 (5.8%)	0	0	0
Ruddy duck, *Oxyura jamaicensis*	46	4 (8.6%)	2 (4.3%)	0	0
Bufflehead, *Bucephala albeola*	35	2 (5.7%)	0	0	0
Canada goose, *Branta canadensis*	148	2 (1.3%)	2 (1.3%)	0	1
Cackling goose, *Branta hutchinsii*	33	1 (3.03%)	0	0	0
Lesser scaup, *Aythya affinis*	14	1 (7.1%)	0	0	0
Ring-necked duck, *Aythya collaris*	65	1 (1.5%)	0	0	0
Common goldeneye, *Bucephala clangula*	39	0	0	1 (2.5%)	0
All other species sampled	76	0	0	0	0
Total	4729	469	113	55	63

Results shown as total positive and percent positive.

**Table 3:** Total number of free ranging wild birds found dead in Washington State with specimens submitted and tested for highly pathogenic avian influenza virus between December 1, 2014 and February 28, 2015.

**Species**	**HPAIV Positive**	**Carcass**	**Swab Only**	**Total**
Canada goose, *Branta Canadensis*	1	1	0	1
Cackling goose, *Branta hutchinsii*		1	0	1
Mallard, *Anas platyrhynchos*	2	33	0	33
Northern shoveler, *Anas clypeata*		1	0	1
American wigeon, *Anas americana*	2	10	1	11
Northern pintail, *Anas acuta*	1*	2	0	2
Bufflehead, *Bucephala albeola*		1	0	1
Barrow’s goldeneye, Bucephala islandica		1	0	1
Common goldeneye, *Bucephala clangula*		6	0	6
Trumpeter swan, *Cygnus buccinator*		3	36	39
Tundra swan, *Cygnus columbianus*		1	0	1
Double-crested cormorant, *Phalacrocorax auritus*		1	0	1
Bald eagle, *Haliaeetus leucocephalus*		9	0	9
Sharp-shinned hawk, *Accipiter striatus*		4	0	4
Cooper’s hawk, *Accipiter cooperii*	1	8	0	8
Red-tailed hawk, *Buteo jamaicensis*	2	5	0	5
Peregrine falcon, *Falco peregrinus*	1	1	0	1
Marbled murrelet, *Brachyramphus marmoratus*		1	0	1
Cassin’s auklet, *Ptychoramphus aleuticus*		7	0	7
Mew gull, *Larus canis*		0	2	2
Glaucous-winged gull, *Larus glaucescens*		0	2	2
Great Horned owl, *Bubo virginianus*		2	0	2
Barred owl, *Strix varia*		3	0	3
Long-eared owl, *Asio otus*		3	0	3
American crow, *Corvus brachyrhynchos*		4	0	4
Crow, Unidentified, *Corvus* species		1	0	1
Total	10	109	41	150

^*^Ip *et al*.^17^.

**Table 4:** Avian species detected with highly pathogenic avian influenza virus (HPAIV) by avian morbidity and mortality surveillance in Washington, December 1, 2014-February 28, 2015.

**Species**	**County**	**Date Found Dead**	**HPAIV Detected**
Mallard, *Anas platyrhynchos*	Whatcom	12/8/2014	HPAIV H5N2
Northern pintail, Anas acuta*	Whatcom	12/8/2014	HPAIV H5N2
American wigeon, *Anas americana*	Whatcom	12/16/2014	HPAIV H5N8
American wigeon, *Anas americana*	Whatcom	12/16/2014	HPAIV H5N8
Mallard, *Anas platyrhynchos*	Whatcom	12/23/2014	HPAIV H5N2
Peregrine falcon, *Falco peregrinus*	Grays Harbor	12/29/2014	HPAIV H5N8
Cooper’s hawk, *Accipiter cooperii*	Whatcom	12/29/2014	HPAIV H5N2
Canada goose, *Branta canadensis*	Jefferson	12/30/2014	HPAIV H5N2
Red-tailed hawk, *Buteo jamaicensis*	Benton	12/31/2014	HPAIV H5N2
Red-tailed hawk, *Buteo jamaicensis*	Skagit	1/9/2015	HPAIV H5N2

^*^Ip *et al*.^17^.
